# Acute Myeloid Leukemia Presenting as Common Colds: An Uncommon Consideration

**DOI:** 10.7759/cureus.53217

**Published:** 2024-01-30

**Authors:** Thomas F Fusillo, Scott Millman, Kamal Menghrajani

**Affiliations:** 1 Internal Medicine, Icahn School of Medicine at Mount Sinai, New York, USA; 2 Leukemia, Memorial Sloan Kettering Cancer Center, New York, USA

**Keywords:** common colds, hematopoietic stem cell transplant, cytarabine, oncology, bone marrow, acute myeloid leukemia (aml), leukemia

## Abstract

Acute myeloid leukemia is the most common form of leukemia and can present with a wide variety of signs and symptoms. This article presents a case of a middle-aged male who presented with ongoing upper respiratory cold-like symptoms and was then found to be severely pancytopenic. A diagnosis of acute myeloid leukemia was made after a bone marrow biopsy, and the patient underwent induction chemotherapy. This article brings to light the uncommon diagnosis of acute myeloid leukemia, from a common presentation, a common cold. Additionally, it discusses the initial workup and diagnostic process of acute myeloid leukemia, risk stratification, and a basic treatment algorithm.

## Introduction

Leukemias are the 10th most common cancer in the adult population, and among all leukemias, acute myeloid leukemia (AML) is the most common, accounting for approximately 80% of all cases [1]. Despite these statistics, AML accounts for only 1% of all cancers in the United States; thus, some clinicians may not be familiar with the presentation and initial workup [2]. The average age of onset for AML is 66 years old, and the prognosis is highly dependent on the patient’s age at the time of diagnosis. Five-year survival rates hover around 10% for those diagnosed after age 60, but approach greater than 50% if diagnosed before 60 [3]. Workup of a new AML case is often left to the specialists but is beneficial even for the generalist to understand. This article discusses a case of new AML presenting as a common cold and explains the initial workup and treatment options.

## Case presentation

A male in his early 50s presented to his primary care physician after several days of cold-like symptoms, extreme weakness that limited walking to just a few minutes before needing to rest, generalized fatigue, and facial pallor. His family all had similar but less severe cold symptoms several days earlier, which resolved for them without issue. He denied any unusual bleeding or bruising. He has no prior medical history and takes no prescription medications. He had an appendectomy and cholecystectomy decades prior. He has seasonal allergies which he takes various over-the-counter antihistamines for as needed. He has never smoked, lives at home with his wife and three children, drinks alcohol socially, and works in construction. He has three healthy siblings, his father died of natural causes, and his mother died from breast cancer.

**Figure 1:**
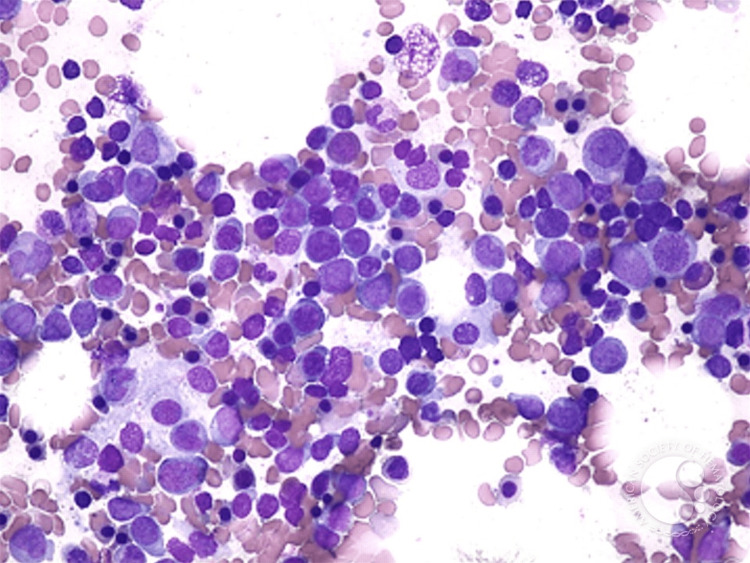
Bone marrow aspirate revealing acute myeloid leukemia Trilineage dysplasia with numerous blasts seen with Romanowsky staining at 400x magnification [4].

Upon initial exam, the patient was immediately sent to the nearest emergency room due to the clinical severity of his weakness and fatigue. In the emergency room, he was found to be pancytopenic, with the labs shown in Table 1. The patient was admitted and received multiple transfusions of packed red blood cells (RBCs). A bone marrow biopsy with core and aspirate was performed, which revealed hypercellular marrow with myeloid predominant hematopoiesis, marked trilineage dysplasia, and 35% blasts on aspirate (Figure 1). Cytogenetics and molecular testing revealed trisomy 8 and no *FLT3*-ITD or *TP53* mutations. A diagnosis of acute myeloid leukemia was made.

**Table 1:** Initial laboratory findings

Lab	Value	Reference Range
White Blood Cells (WBC)	2,300 cells/mm^3^	4,500-11,000 cells/mm^3^
Hemoglobin (HGB)	3.4 g/dL	11.7-15.0 g/dL
Platelets (PLT)	47,000/mm^3^	150,000-450,000/mm^3^
Absolute Neutrophil Count (ANC)	166/uL	1,900-8,000/uL
Peripheral Blasts	2%	0-2%

The patient was quickly started on induction “seven plus three” chemotherapy with cytarabine and daunorubicin. This was complicated by neutropenic fevers due to *Serratia* bacteremia, which was treated with piperacillin/tazobactam. He was also started on acyclovir and posaconazole for antimicrobial prophylaxis while neutropenic (ANC less than 500 neutrophils per microliter). Chromosomal analysis once again revealed an abnormal karyotype in 20 metaphase cells with a gain of chromosome eight. No *FLT3*-ITD, TKD, or *TP53* mutations were detected. The patient tolerated induction therapy well and a day 14 mid-cycle bone marrow biopsy revealed a markedly hypocellular marrow with few AML blasts (less than 5%).

A day 37 recovery marrow revealed a hypercellular marrow with 12% blasts on aspirate. The myeloid lineage was dominant, indicating continued AML disease. Additionally, the original trisomy eight was present. However, *RUNX1* and *JAK2* mutations were now detected as well. This classified the patient as high/poor risk, and he proceeded to reinduction with fludarabine, cytarabine, and G-CSF (FLAG)-based regimen with the goal to achieve morphological complete remission and proceed to hematopoietic stem cell transplantation (HSCT).

## Discussion

Acute myeloid leukemia is a rare but serious cancer, affecting over 20,000 Americans a year, the majority being adults [2]. The disease is characterized by clonal disorder of the hematopoietic stem or progenitor cells, often down to the level of the myeloblasts [5]. This is often complicated by additional chromosomal, cytogenetic, or molecular abnormalities. The majority of AML cases present various signs of cytopenias: shortness of breath from anemia, easy bruising from thrombocytopenia, or infections from neutropenia/leukopenia. However, there are also several hematologic emergencies associated with AML, which could be the presenting symptoms. These include coagulopathies such as disseminated intravascular coagulation (DIC), tumor lysis syndrome (TLS), and leukostasis [5].

Initial evaluation includes a CBC, which often shows low hemoglobin and platelets. WBC can be variable, with approximately 50% of patients having a normal or decreased WBC count and 20% having a WBC of greater than 100 k/uL [5]. Therefore, pancytopenia is a highly notable finding and can help distinguish symptom etiology if AML is presenting as a viral illness, which may otherwise present with low WBC count but normal hemoglobin and platelets [5]. The hallmark finding of AML is blasts, either in the periphery or in the marrow. Further workup includes three major parts: bone marrow biopsy to assess morphology, cytogenetics with karyotype analysis, and molecular pathology. Recent advancements in leukemia cytogenetics and diagnostic molecular pathology have allowed for a more tailored risk stratification and treatment approach (Table 2) [6-8].

**Table 2:** AML risk status based on cytogenetics and molecular abnormalities Complex karyotype refers to three or more chromosomal abnormalities [6-8].

Risk Status	Cytogenetics	Molecular
Low/Favorable	inv(16), t(16;16), t(8;21), t(15;17)	*NPM1* (*FLT3*-ITD neg.), *CEBPA*
Intermediate	Normal cytogenetics, trisomy 8, t(9;11)	-
High/Poor	Complex karyotype, -5/5q-, -7/7q-, 11q23-, inv(3), t(3;3), t(6;9), t(9;22)	*TP53*, *RUNX1*, *ASXL1*, *FLT3*-ITD

**Figure 2:**
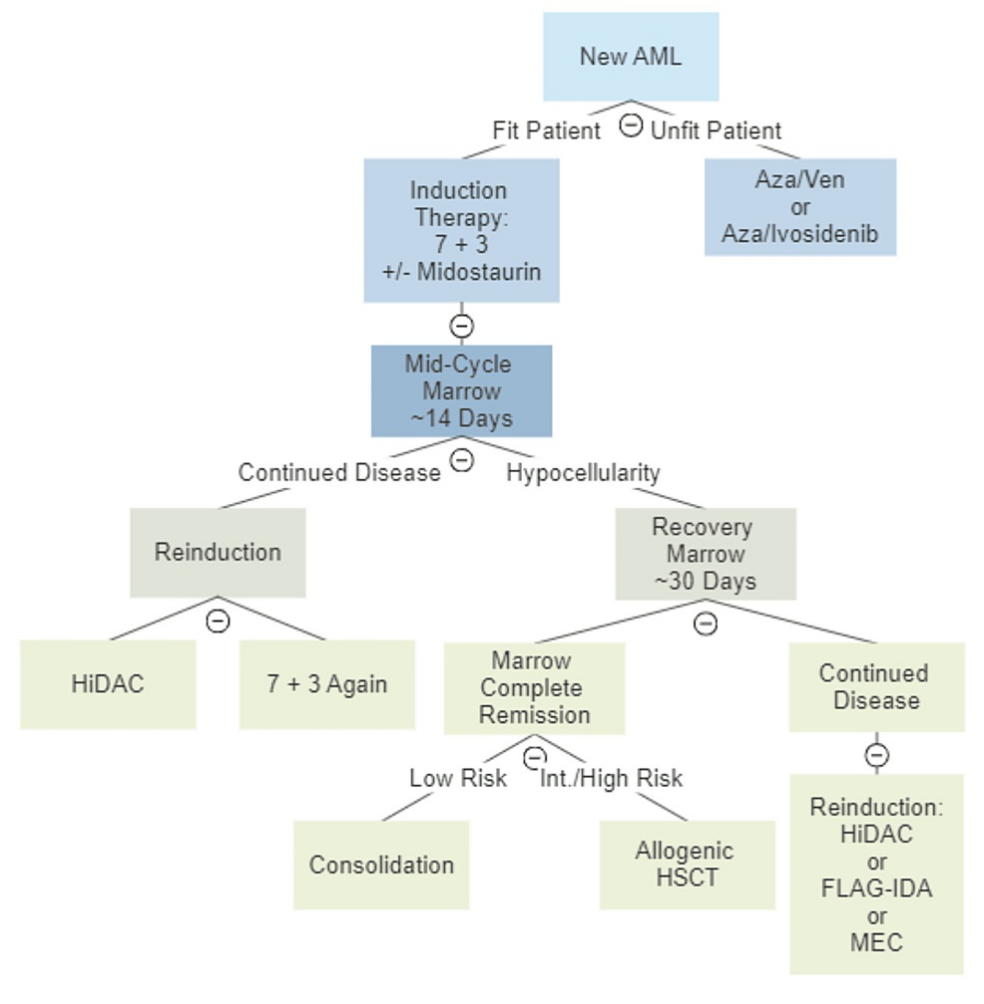
Newly diagnosed AML treatment flowchart Azacitidine (Aza); venetoclax (ven); 7 + 3 refers to seven days of cytarabine with three concurrent days of an anthracycline; high-dose cytarabine (HiDAC); hematopoietic stem cell transplant (HSCT); fludarabine cytarabine idarubicin and G-CSF (FLAG-IDA); mitoxantrone etoposide cytarabine (MEC).

The first step in starting treatment is determining the fitness of the patient. Fit and younger patients are generally started on intensive induction therapy, while older and unfit patients are started with low-intensity therapies (Figure 2). Fitness refers to the patient’s ability to tolerate intensive chemotherapy and is often correlated with age. The mainstay of induction therapy is cytarabine and an anthracycline, typically daunorubicin or idarubicin, known as “seven plus three.” This comprises seven days of cytarabine with three concurrent days of anthracycline. Alternatively, for the unfit patient, the general mainstay of treatment is azacitidine plus venetoclax (aza/ven). Additionally, there are now multiple targeted therapies such as midostaurin and ivosidenib that target specific gene mutations such as FLT3 and IDH [3,6,9]. However, it is important to note that different cancer centers may treat AML with slightly different regimens. For example, MD Anderson uses idarubicin, high-dose cytarabine, and an adenosine nucleoside analog such as fludarabine (FAI/FLAG-IDA) or cladribine (CLIA) as induction therapy for fit patients, while Memorial Sloan Ketter Cancer Center uses standard dose cytarabine and daunorubicin or idarubicin (seven plus three) [9,10].

Intensive induction therapy is followed by a precipitous drop in all three cell lines, which highlights the importance of infection prophylaxis during this time. The cytopenic nadir generally comes approximately two weeks after beginning induction therapy, hence the recommendation to obtain a “mid-cycle” bone marrow biopsy around day 14. Importantly, the timing of this biopsy should be based on the cell counts, not solely on the time since induction was initiated [11]. The purpose of the mid-cycle marrow is to evaluate the success of marrow ablation or the need for further chemotherapy [6,9]. If the mid-cycle marrow reveals continued disease, reinduction might be necessary and is often done by repeating seven plus three or using high-dose cytarabine (HiDAC) [9].

If the mid-cycle marrow reveals a pronounced hypocellularity, the patient continues to be monitored until a recovery marrow is performed. The recovery marrow is usually performed around 30 days after initiating induction therapy, and its purpose is to evaluate the morphology and phenotype of the recovering bone marrow. Similar to the mid-cycle marrow, it is more important to do this when all three cell lines begin to recover rather than on day 30 regardless of counts. If the recovery marrow reveals marrow/morphological complete remission (MCR), the patient will proceed to either consolidation or HSCT depending on the risk stratification. However, if the recovery marrow reveals continued disease, the patient will often proceed to reinduction. This can be done with the same regimen as the original induction, or alternatives such as HiDAC, FLAG-IDA, or mitoxantrone, etoposide, and cytarabine (MEC).

## Conclusions

Acute myeloid leukemia is a relatively uncommon disease with broad ramifications. Although the disease is almost always treated by oncologists and specialists, the initial diagnosis often starts with the primary care physician or general practitioner. Thus, the variety of ways AML can initially present is important for organizing a thorough differential. Overall, this case brought to light one of these many ways AML can present, via common cold symptoms, and the general workup and management of such a case.

## References

[1] Vakiti A, Mewawalla P (2023). Acute myeloid leukemia. StatPearls.

[2] (2024). American Cancer Society: Key statistics for acute myeloid leukemia (AML). https://www.cancer.org/cancer/types/acute-myeloid-leukemia/about/key-statistics.html.

[3] Roloff GW, Odenike O, Bajel A, Wei AH, Foley N, Uy GL (2022). Contemporary approach to acute myeloid leukemia therapy in 2022. Am Soc Clin Oncol Educ Book.

[4] Maslak P (2024). Acute myeloid leukemia with multilineage dysplasia - 2. American Society of hematology.

[5] Goldberg AD, Tallman MS (2023). Acute Myeloid Leukemia (AML). Pocket Oncology.

[6] Pollyea DA, Bixby D, Perl A (2021). NCCN Guidelines insights: acute myeloid leukemia, version 2.2021. J Natl Compr Canc Netw.

[7] Papaemmanuil E, Gerstung M, Bullinger L (2016). Genomic classification and prognosis in acute myeloid leukemia. N Engl J Med.

[8] Grimwade D, Hills RK, Moorman AV (2010). Refinement of cytogenetic classification in acute myeloid leukemia: determination of prognostic significance of rare recurring chromosomal abnormalities among 5876 younger adult patients treated in the United Kingdom Medical Research Council trials. Blood.

[9] Kantarjian H, Kadia T, DiNardo C (2021). Acute myeloid leukemia: current progress and future directions. Blood Cancer J.

[10] (2024). Treatment for acute myeloid leukemia (AML). https://www.mskcc.org/cancer-care/types/leukemias/treatment/acute-myeloid-leukemia.

[11] Morris TA, DeCastro CM, Diehl LF (2013). Re-induction therapy decisions based on day 14 bone marrow biopsy in acute myeloid leukemia. Leuk Res.

